# Reactive Oxygen Species in Stem Cells

**DOI:** 10.1155/2015/159080

**Published:** 2015-07-29

**Authors:** Tullia Maraldi, Cristina Angeloni, Elisa Giannoni, Christian Sell

**Affiliations:** ^1^Department of Surgical, Medical, Dental and Morphological Sciences with interest in Transplant, Oncology and Regenerative Medicine, University of Modena and Reggio Emilia, Via del Pozzo 71, 41124 Modena, Italy; ^2^Department for Life Quality Studies, Alma Mater Studiorum University of Bologna, Via Irnerio 48, 40126 Bologna, Italy; ^3^Department of Experimental and Clinical Biomedical Sciences, University of Florence, Viale Morgagni 50, 50134 Florence, Italy; ^4^Department of Pathology, Drexel University College of Medicine, 15th Street MS 435, Philadelphia, PA 19102, USA

Stem cells are defined by their unique ability to self-renew and their multipotent differentiation capacity, thus maintaining tissue homeostasis throughout the life of a multicellular organism. Stem cells reside in niches characterized by hypoxia and low reactive oxygen species (ROS), both of which are critical for maintaining the potential for self-renewal and stemness. Until recently, the focus in stem cell biology has been on the adverse effects of ROS, particularly the damaging effects of ROS accumulation on tissue aging and the development of cancer. However, it has become increasingly clear that, in some cases, redox status plays an important role in stem cell maintenance, that is, regulation of the cell cycle. In fact, ROS at low levels function as signaling molecules to mediate cell proliferation, migration, and differentiation and gene expression. ROS levels in stem and progenitor cells have a clear correlation with cellular functions and are regulated by a fine-tuning of the balance between ROS-generating and antioxidant defense systems.

This special issue tries to fully decipher the underlying molecular mechanisms involved in the maintenance of stem cell self-renewal, which is critical to address the important role of redox homeostasis in the regulation of both self-renewal and differentiation of stem cells.

The safe use of stem cells for clinical applications still requires culture improvements to obtain functional cells. The review of S. Sart et al. investigates the roles of ROS in the maintenance of self-renewal, proliferation, and differentiation of mesenchymal stem cells (MSCs) and pluripotent stem cells (PSCs) and highlights that the tight control of stem cell microenvironment, including cell organization, metabolic, and mechanical environments, may be an effective approach to regulate endogenous ROS generation. While high levels of ROS have detrimental effects on stem cells through the induction of oxidative stress, physiological levels of ROS play an important role in the regulation of stem cell fate decision. Mild levels of ROS act as secondary messengers by interfering with various signaling pathways that regulate stem cell proliferation, survival, and differentiation. However, the contribution of the specific site of ROS production and the specific type of ROS to the regulation of stem cell fate requires further delineation. In addition, the exact threshold levels of ROS discerning between the role as damaging molecules or as enhancers of stem cell signaling pathway are not clearly defined. Combined with accurate ROS measurement, regulation of biochemical and biomechanical environment of stem cells to modulate redox status can lead to the controlled proliferation and differentiation of stem cells towards various biomedical applications.

Regarding the site of ROS production and the damaging role of ROS in human stem cells the article of T. Maraldi et al. suggests a particular role of NAD(P)H oxidase enzyme family. In this study they show that Nox4 nuclear expression depends on the donor and it correlates with the expression of redox transcription factors involved in stemness regulation and with the differentiation potential. Taken together, these results suggest that nuclear Nox4 regulation may have important effects on stem cell capability through modulation of transcription factors and DNA damage and the extensive donor-to-donor heterogeneity.

The study of O. G. Lyublinskaya et al. focuses on the involvement of ROS in the process of MSCs “waking up” and entering the cell cycle after the quiescence. Using human endometrial MSCs, they showed that intracellular basal ROS levels are positively correlated with the proliferative status of the cell cultures. In fact, they observed that physiologically relevant levels of ROS are required for the initiation of human MSC proliferation and that low levels of ROS due to the antioxidant treatment can block the stem cell self-renewal.

Despite many attractive advantages of MSCs, replicative senescence of MSCs occurs, eventually decreasing their functional capacities. MSC aging is proposed to potentially contribute to organism aging leading to loss of tissue homeostasis. It is essential to consider the role of ROS in the aging process of MSCs since MSCs and their regenerative potential have revealed a critical therapeutic approach to aging. In lung diseases, the excessive amount of ROS from the ambient air might be associated with respiratory failure through unsuspected mechanisms that regulate declines in the residual stem cell function with age. However, the review of S.-R. Yang concludes that much work remains to be done to understand the ROS-mediated mechanisms that regulate residual stem cells. Elucidating these mechanisms will be critical to understand how stem cell based or antioxidant therapies are effective in certain tissues including lungs.

Regarding the role of ROS in the differentiation process the article of S. Kostyuk et al. describes that GC-rich fragments in the pool of cell-free DNA (cfDNA), circulating throughout the bloodstream of both healthy people and patients with various diseases, can potentially induce oxidative stress and DNA damage response and affect the direction of MSCs differentiation in human adipose-derived MSCs. Such a response may be one of the causes of obesity or osteoporosis.

Similar to normal stem cells, cancer stem cells (CSCs) also show lower intracellular ROS levels, suggesting that maintenance of a reduced intracellular environment is associated with an undifferentiated state. CSCs are responsible for cancer recurrence after chemotherapy or radiotherapy. However, the role of ROS in CSCs remains poorly understood and intensive research is desperately needed.

The review of S. Ding focuses on ROS generation and removal in CSCs and their effects on CSC self-renewal and differentiation through the modulating of ROS-dependent cellular processes. While information on ROS regulation in CSCs are limited, there is fast emerging evidence that ROS may play essential role in the self-renew and differentiation ability of CSCs. The review concludes that targeting CSCs via ROS and antioxidant enzymes regulation holds a great promise in cancer therapy.

Similarly, the review of J. Liu and Z. Wang analyses the particular role of ROS in CSCs. Increasing evidence proposes that a low concentration of ROS can maintain the stemness of CSCs and contribute to tumorigenesis and development. Various systems exist for the regulation of ROS in CSCs. For example, under hypoxic environment, increased levels of ROS induce the expression of HIFs in CSCs, resulting in the upregulation of CSC markers and the reduction of intracellular ROS level and thus facilitating CSCs survival and proliferation. Consequently, the induction of oxidative stress appears to be a promising approach for the preferential killing of cancer cells, including CSCs. In addition, a more detailed investigation of therapies with direct or indirect effects on ROS will help to define a made-to-order therapeutic schedule with a lower tendency toward promoting the development of resistance to treatment.

*Tullia Maraldi* *Tullia Maraldi* *Cristina Angeloni* *Cristina Angeloni* *Elisa Giannoni* *Elisa Giannoni* *Christian Sell* *Christian Sell*

